# Resistant Maltodextrin Ameliorates Altered Hepatic Lipid Homeostasis via Activation of AMP-Activated Protein Kinase in a High-Fat Diet-Fed Rat Model

**DOI:** 10.3390/nu11020291

**Published:** 2019-01-29

**Authors:** Shing-Hwa Liu, Chen-Yuan Chiu, Lin-Hui Huang, Meng-Tsan Chiang

**Affiliations:** 1 Graduate Institute of Toxicology, College of Medicine, National Taiwan University, Taipei 10051, Taiwan; shinghwaliu@ntu.edu.tw; 2 Department of Pediatrics, College of Medicine, National Taiwan University Hospital, Taipei 10041, Taiwan; 3 Department of Medical Research, China Medical University Hospital, China Medical University, Taichung 40402, Taiwan; 4 Institute of Food Safety and Health, College of Public Health, National Taiwan University, Taipei 10055, Taiwan; kidchiou@gmail.com; 5 Department of Food Science, College of Life Science, National Taiwan Ocean University, Keelung 20224, Taiwan; vivian81050811@gmail.com

**Keywords:** resistant maltodextrin, high-fat diet, fatty liver, hepatic lipid metabolism, AMP-activated protein kinase

## Abstract

Many studies have shown that resistant maltodextrin (RMD) possesses blood cholesterol lowering and anti-obesity effects. In order to investigate the effect of RMD on lipid metabolism in the liver, rats were fed with a high-fat (HF) diet for 7 weeks to induce hyperlipidemia and fatty liver. Normal control rats were fed with a normal diet. HF-diet-fed rats were treated with 5% RMD for 8 weeks. The results showed that the increased plasma aspartate aminotransferase (AST) and alanine aminotransferase (ALT) activities, the increased hepatic triglyceride and total cholesterol levels, and fatty liver in HF-diet-fed rats were significantly decreased after supplementation with RMD. Supplementation with RMD significantly (1) induced AMP-activated protein kinase (AMPK) phosphorylation; (2) inhibited the activities of acetyl-CoA carboxylase (ACC), fatty acid synthase (FAS), and HMG-CoA reductase (HMGCR); (3) suppressed the protein expression of peroxisome proliferator activated receptor (PPAR)-γ; (4) increased β-oxidation of fatty acids by increasing the protein expression carnitine palmitoyl transferase 1α (CPT-1α) in the livers of HF-diet-fed rats. Taken together, supplementation of RMD was capable of inhibiting lipogenic enzyme activities and inducing fatty acid β-oxidation through increasing AMPK activation, thereby reducing lipid accumulation in the liver.

## 1. Introduction

Lack of physical activity and excessive calorie intake creates an energy imbalance in the human body. Sugars and lipids in the diet are metabolized in the body to triglyceride (TG) and accumulate in the adipose tissue and liver, which may cause obesity and fatty liver. Non-alcoholic fatty liver disease (NAFLD) is a chronic liver disease, which is characterized by hepatic lipid accumulation. The prevalence of NAFLD in the adult population in Taiwan is as high as 33.6% [1]. The causes of NAFLD include drugs, obesity, diabetes, and hyperlipidemia to induce non-alcohol liver injury, which may eventually evolve into non-alcoholic steatohepatitis (NASH), liver fibrosis, liver cirrhosis, and finally liver failure or liver cancer [2].

To explore better remedies with fewer side effects and higher potency for liver injuries, bioactive ingredients from natural products have recently been well applied and used as food or dietary supplements to improve liver injuries by their antioxidant and anti-hyperlipidemia properties [3,4,5]. In turn, explored bioactive compounds or synthetic supplements have been related to adjunctive therapy in subjects with metabolic syndrome and NAFLD. Therefore, it has been a critical issue to focus on the development of innovative beneficial targets for functional foods or food supplements against liver injuries. Resistant maltodextrin (RMD) is a soluble dietary fiber that has been confirmed as “generally recognized as safe” (GRAS) by US FDA. Fibersol-2^®^, an RMD with about 2000 Da molecular weight, contains 90% dietary fiber and provides 0.02 g sugar/g and 1.6 kcal/g. It can be obtained by hydrolyzing corn starch with hydrochloric acid, and hydrolyzing with α-amylase and glucoamylase [6]. Kishimoto et al. (2000) have found that RMD significantly reduced serum total cholesterol (TC) and TG and body fat accumulation, especially visceral fat, in a clinical trial [7]. RMD has also been shown to reduce waist circumference, visceral fat area, insulin resistance, and serum TG in subjects with metabolic syndrome [8]. However, the effect and mechanism of RMD on hepatic lipid metabolism and fatty liver still remain to be clarified. To assess possible effects and mechanisms of RMD on lipid metabolism in the liver, we investigated the long-term effects of diets supplemented with RMD on a HF-diet-fed Sprague–Dawley rat model. Moreover, orlistat, a potent inhibitor of gastric and pancreatic lipase which acts by reducing dietary fat absorption [9], was used as a positive control in this study. Orlistat has recently been suggested as a beneficial therapy for NAFLD [10].

## 2. Materials and Methods

### 2.1. Experimental Animals

Male Sprague–Dawley rats (6 weeks old) were obtained from BioLASCO (Taipei, Taiwan). Rats were acclimatized for one week (body weight: 183.6 ± 7.0 g, *n* = 32). Rats were fed with chow diet (Rodent Laboratory Chow, Ralston Purina, St. Louis, MO, USA). Twenty four rats were fed with an HF diet for 7 weeks to induce hyperlipidemia and fatty liver. Eight rats were fed with a normal diet (normal control diet group; NC). The HF-diet-fed rats were then treated with or without 0.2% orlistat (HO) or 5% RMD (Fibersol-2) (FS) (*n* = 8 of each group) for 8 weeks. The diet composition of each group is listed in Table 1. Rats were housed in stainless steel cages with 40–60% relative humidity, 23 ± 1 °C, and a 12 h light/dark cycle. All animals were fed ad libitum. At the end of the experimental intervention, rats were euthanized under anesthesia after 12 h fasting. Blood, liver, and adipose tissues were collected. Feces were collected 3 consecutive days before euthanasia. The Animal House Management Committee of the National Taiwan Ocean University approved this animal study (Permission number: 105009). The procedures were performed according to the guidelines for care and use of laboratory animals [11]. Orlistat (Zerocal^®^) was purchased from Taiheshuo Pharmaceutical Technology Co., Ltd. (Taipei, Taiwan). RMD (Fibersol-2^®^) was purchased from Matsutani Chemical Industry (Hyogo, Japan). Body weight and food consumption were recorded once a week, and the changes of body weight and food consumption were represented as mean ± S.D. (standard deviation) for each group of rats. Feed efficiency was calculated by the following equation: (body weight gain (g)/food intake (g/day)) × 100%. The dose for RMD was selected according to previous studies [12,13].

### 2.2. Liver Lipid Extraction

The liver lipid extraction was performed as previously described by Folch et al. (1957) [14], with a minor modification. The liver tissue (0.2 g) was homogenized with the chloroform/methanol (2:1, *v*/*v*) mixture (4 mL), and then centrifuged at 3000 rpm for 10 min at 4 °C. The samples of liver lipids were stored at −70 °C until analysis.

### 2.3. Detection of Triglyceride (TG), Cholesterol (TC), and Lipoprotein Levels, and Aspartate Aminotransferase (AST) and Alanine Aminotransferase (ALT) Activity

The TG and TC levels in liver and blood were detected by assay kits (Audit Diagnostics, Cork, Ireland). The absorbance at 500 nm was measured. The plasma low-density lipoprotein (LDL), high-density lipoprotein (HDL), and very-low-density lipoprotein (VLDL) were isolated through a density gradient determined by an ultracentrifuge (Hitachi, Tokyo, Japan) with 194,000× *g* at 10 ℃ for 3 h, and then collected by tube slicing. The AST and ALT activities were determined by AST and ALT enzymatic assay kits (Randox, Antrim, UK). The absorbance was detected at 340 nm.

### 2.4. Measurement of Hepatic Acetyl-CoA Carboxylase Activity (ACC)

The reaction mixture, containing 50 mM Tris-HCl buffer, 10 mM MgCl_2_, 10 mM potassium citrate, 3.75 mM glutathione, 12.5 mM KHCO_3_, 0.675 mM BSA, 0.125 mM acetyl-CoA, 3.75 mM ATP, liver cytosol preparations, and 10 mM NADPH, was added to 96 well microplates and mixed with a shaker. The absorbance changes were measured at 340 nm using an ELISA reader (Multiskan Go1510, Thermo Fisher Scientific, Waltham, MA, USA). 

### 2.5. Measurement of Hepatic Fatty Acid Synthase Activity (FAS)

The reaction mixture, containing 0.2 M K_2_HPO_4_ buffer, 20 mM dithiothreitol (DTT), 0.25 mM acetyl-CoA, 60 mM EDTA⋅2Na, 0.39 mM malonyl-CoA, liver cytosol preparations, and 6 mM NADPH, was added to 96 well microplates and mixed with a shaker. The absorbance changes were measured at 340 nm using an ELISA reader (Thermo Fisher Scientific).

### 2.6. Measurement of Hepatic HMG-CoA Reductase (HMGCR) Activity

The reaction mixture, containing 0.2 M KCl, 0.16 M KH_2_PO_4_, 0.004 M EDTA, 0.01 M DTT, 0.1 mM HMG-CoA, liver microsomal preparations, and 0.2 mM NADPH, was added to 96 well microplates and mixed with a shaker. The absorbance changes were measured at 340 nm using an ELISA reader (Thermo Fisher Scientific).

### 2.7. Histological Examination

Hepatic paraffin sections (5 μm) were performed for histological examination. The tissue sections were stained with hematoxylin and eosin (H&E). The images were observed by a microscope equipped with a digital camera. The histopathological scoring was according to “NAFLD activity score” as previously described by Dyson et al. (2014) [15]. Relative vacuolated areas in liver tissues were calculated with the ImageJ 1.48 software (National Institutes of Health, Bethesda, MD, USA). 

### 2.8. Protein Expression Analysis

Protein expression analysis was performed by Western blotting, as previously described [16]. Protein extracts (50–100 μg) were added into 8% or 10% SDS-PAGE gel, and then transferred to polyvinylidene difluoride (PVDF) membranes (Bio-Rad, Hercules, CA, USA). Membranes were reacted with primary antibodies for AMP-activated protein kinase (AMPK)α and phosphorylated AMPKα (p-AMPKα) (Cell Signaling Technology, Danvers, MA, USA), peroxisome proliferator activated receptor (PPAR)-γ, PPAR-α, carnitine palmitoyl transferase 1α (CPT1-α), and β-actin (Santa Cruz Biotechnology, Santa Cruz, CA, USA) after blocking for 1 h. Membranes were then reacted with secondary antibodies. Cross-reactivity was determined by a Bio-Rad enhanced chemiluminescence kit. The quantification was performed by a densitometric analysis with an ImageJ software (National Institutes of Health, Bethesda, MD, USA).

### 2.9. Statistical Analysis

Data are presented as the mean ± standard deviation (S.D.). The statistical difference was evaluated by one-way analysis of variance (ANOVA) followed by Dunnett’s test using an IBM SPSS Statistics 22.0 software. The *p* < 0.05 is considered as statistically significant difference.

## 3. Results

### 3.1. Effects of RMD on the Changes of Body Weight, Liver Weight, Serum and Liver Lipids, and Liver Histopathology in HF-Diet-Fed Rats

After 7 weeks of induction period, the TC level and AST and ALT activities were significantly higher in the HF group than that in the NC group (TC (mg/dL): NC, 67.8 ± 9.4, *n* = 8, HF, 97.9 ± 48.5, *n* = 24, *p* < 0.05; AST (U/L): NC, 13.2 ± 1.7, *n* = 8, HF, 30.5 ± 23.7, *n* = 24, *p* < 0.05; ALT (U/L): NC, 6.0 ± 2.7, *n* = 8, HF, 11.7 ± 6.4, *n* = 24, *p* < 0.05). The plasma TG level in the HF group was significantly lower than that in the NC group (TG (mg/dL): NC, 116.4 ± 33.4, *n* = 8, HF, 60.6 ± 15.3, *n* = 24, *p* < 0.05). These results indicate that hypercholesterolemia and liver dysfunction were induced in HF-diet-fed rats.

As shown in Table 2, after 8 weeks of supplementation of RMD or orlistat in HF-diet-fed rats, the body weight in the orlistat (HO) group was significantly lower than that of the HF group. There were no statistical differences between RMD alone (FS) and HF groups. The food intake was significantly higher in the NC and HO groups than that of the HF group. The feed efficiency was significantly higher in the HF group than that of the NC group; the feed efficiency was significantly lower in the HO group than in the other groups. The water intake was significantly higher in the HO group than in the other groups. There were no statistical differences among the NC, FS, and HF groups. Moreover, as shown in Table 3, the liver weight of the HF group was significantly higher than that of the NC and HO groups. The adipose tissue weight was significantly lower in the HO group than in the other groups. 

As shown in Table 4, the plasma TC and LDL-C levels and AST and ALT activities were significantly increased, and TG and HDL-C levels and HDL-C/LDL-C ratio were significantly decreased in HF-diet-fed rats. Supplementation of RMD significantly reduced the increased TC and LDL-C levels and AST and ALT activities in HF-diet-fed rats. Orlistat treatment did not affect the changes of plasma lipids, but significantly reduced the increased AST and ALT activities in HF-diet-fed rats. The levels of TC and TG in the livers of the HF group were also significantly higher than those in the NC group (Table 5). Supplementation of RMD or orlistat significantly reduced the increased TC and TG levels in the livers of HF-diet-fed rats (Table 5).

As shown in Table 6, the fecal wet and dry weights were significantly higher in the HO group than in the other groups. The amount of TG, but not TC, excretion in feces was significantly higher in the HO group than in the other groups. However, the amount of TC excretion in feces was significantly higher in the FS group than in the other groups (Table 6).

**Figure 1:**
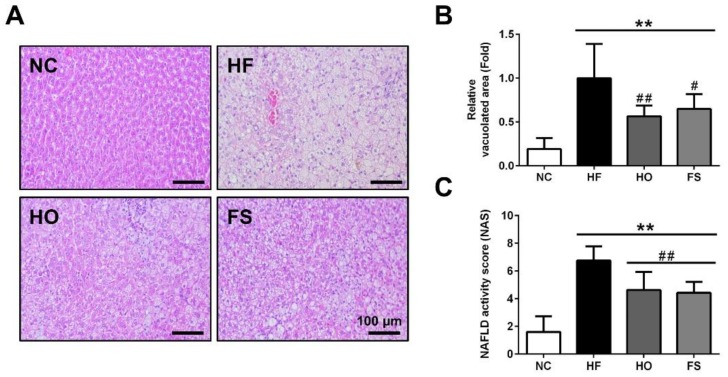
Effects of RMD and orlistat on the changes of hepatic histology in HF-diet-fed rats. Histological analysis in the livers of HF-diet-fed rats treated with or without RMD, and orlistat for 8 weeks was shown. (**A**) Representative hematoxylin and eosin (H&E) stained images of liver tissues were shown. (**B**) Relative vacuolated area and (**C**) NAFLD activity score (NAS) were quantified. Scale bar: 100 μm. * present *p* < 0.05, ** present *p* < 0.01 as compared with NC group; ^#^ present *p* < 0.05, ^##^ present *p* < 0.01 as compared with HF group. NC: normal control diet; HF: high-fat diet; HO: high-fat diet containing 0.2% orlistat; FS: high-fat diet containing 5% Fibersol-2.

As shown in Figure 1, the results of the histological examination of the liver showed a complete hepatocyte pattern and no fat vacuoles in the NC group. In the HF group, large fat vacuoles were observed, the liver cell morphology was obviously destroyed, and the nucleus was squeezed by the fat vacuole to the edge of the liver cells. Supplementation of RMD or orlistat effectively reduced the histopathological changes in the liver of HF-diet-fed rats.

### 3.2. Effects of RMD on the Activities of Acetyl-CoA Carboxylase (ACC), Fatty Acid Synthase (FAS), and HMG-CoA Reductase (HMGCR) and Lipid Metabolism-Related Signaling Molecules in the Liver of HF-Diet-Fed Rats

**Figure 2:**
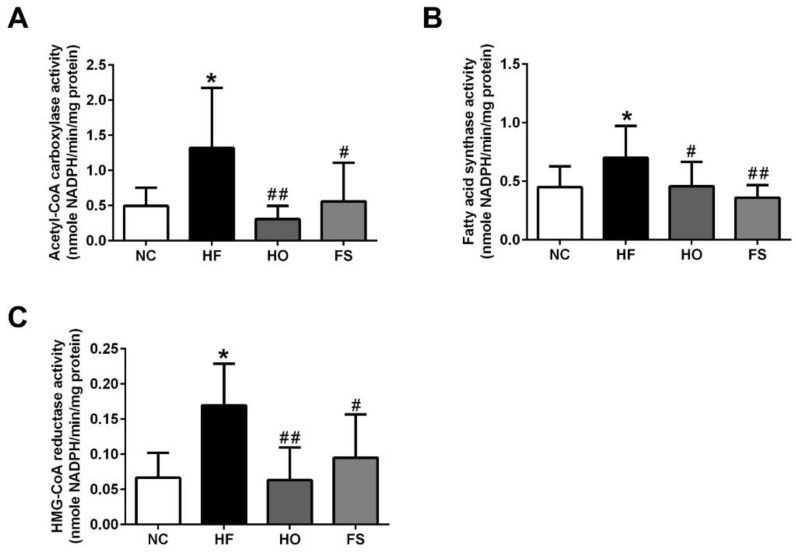
Effects of RMD and orlistat on the activities of acetyl-CoA carboxylase (ACC), fatty acid synthase (FAS), and HMG-CoA reductase (HMGCR) in the livers of HF-diet-fed rats. Enzymatic activities for ACC (**A**), FAS (**B**), and HMGCR (**C**) in the livers of HF-diet-fed rats treated with or without RMD and orlistat for 8 weeks are shown. Results are expressed as mean ± S.D. for each group (*n* = 8). * present *p* < 0.05, ** present *p* < 0.01 as compared with NC group; ^#^ present *p* < 0.05, ^##^ present *p* < 0.01 as compared with HF group. NC: normal control diet; HF: high-fat diet; HO: high-fat diet containing 0.2% orlistat; FS: high-fat diet containing 5% Fibersol-2.

We next investigated the changes of activity for lipogenic enzymes ACC and FAS, and the activity of the cholesterol synthesis rate limiting step enzyme HMGCR in the liver of HF-diet-fed rats with or without treatments of tested materials. As shown in Figure 2, the activities of ACC, FAS, and HMGCR were significantly increased in the livers of HF-diet-fed rats, which could be significantly reversed by the treatment of RMD or orlistat.

**Figure 3:**
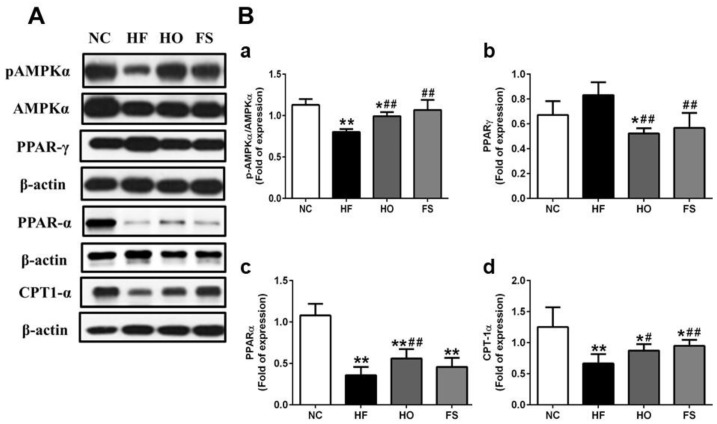
Effects of RMD and orlistat on the protein expression of lipid metabolism-related signaling molecules in the livers of HF-diet-fed rats. Protein expression for pAMPKα/AMPKα, PPAR-α, PPAR-γ, and CPT-1α in the livers of HF-diet-fed rats treated with or without RMD and orlistat for 8 weeks are shown (**A**). Protein expression was determined by Western blotting. Densitometric analysis for protein levels corrected to each internal control is shown (**B**). Results are expressed as mean ± S.D. for each group (*n* = 4–6). * present *p* < 0.05, ** present *p* < 0.01 as compared with NC group; ^#^ present *p* < 0.05, ^##^ present *p* < 0.01 as compared with HF group. NC: normal control diet; HF: high-fat diet; HO: high-fat diet containing 0.2% orlistat; FS: high-fat diet containing 5% Fibersol-2.

We further tested the changes of lipid metabolism-related signaling molecules in the livers of HF-diet-fed rats with or without treatments of tested materials. The protein expression of phosphorylated AMPK was significantly decreased in the liver of HF-diet-fed rats, which could be reversed by the treatment of RMD or orlistat (Figure 3A,B-a). The protein expression of PPAR-γ in the liver was significantly higher in the HF group than in the HO and FS groups, although there was no markedly significant difference between control and HF groups (Figure 3A,B-b). The expression of PPAR-α protein in the liver was significantly lower in the HF group than in the other groups, although there was no significant difference between the HF and FS groups (Figure 3A,B-c). The protein expression of CPT1-α in the liver was significantly lower in the HF group than in the other groups, although there was no significant difference between the HF and HO groups (Figure 3A,B-d). These results indicate that RMD is capable of improving the alterations of lipogenic enzymes and lipid metabolism-related signaling molecules in the livers of HF-diet-fed rats.

## 4. Discussion

In this study, we demonstrate for the first time that supplementation of RMD effectively reduces TC and TG accumulation, inhibits lipogenic enzyme activities, induces fatty acid β-oxidation, and improves steatosis in the livers of HF-diet-fed rats via the AMPK-related signals. 

The present work shows that the HF group had significantly lower food intake than the NC group. It was consistent with the previous reports that a reduced food intake in HF-diet-fed rats compared to control rats was also observed [17,18]. Orlistat significantly reduced body weight in HF-diet-fed rats after 8 weeks of treatment. It is speculated that orlistat may block the absorption of dietary fat, resulting in an increase in food intake due to insufficient caloric intake. Borovicka et al. (2000) have found that the intestinal lipolytic enzyme activity could be markedly inhibited after administration of orlistat to healthy subjects [19]. In addition, orlistat might affect the function of the salivary gland, accelerate gastric emptying, reduce gastric pyloric pressure, and increase duodenal motility to cause an increase in food intake in rats [20]. Supplementation of RMD did not affect the body weight in HF-diet-fed rats after 8 weeks of treatment.

Increased plasma TC level and the decreased plasma TG level were observed in rats fed with the HF diet. The accumulation of TG in the liver may result in a decrease in blood level of TG. It has been shown that the decrease of plasma TG level in mice under HF diet feeding may be due to lower carbohydrate intake [21]. In addition, the putative mechanism of this paradoxical result may be correlated to several mediators contributing to the results of TG accumulation in the liver and the decrease of plasma TG, including the downregulation of microsomal angiopoietin-like 4 (an inhibitor of plasma lipoprotein lipase), triglyceride transfer protein (a hepatic TG transporter), and apolipoprotein E (a hepatic VLDL-TG secretion enhancer) under the HF-diet-fed condition, which is consistent with our previous studies [18,22] and other studies [21,23,24]. Supplementation of RMD significantly reduced the increased plasma TC in HF-diet-fed rats, which was consistent with previous findings [12,25]. We also found that the blood AST and ALT activities, liver TC and TG levels, and liver lipid accumulation were significantly increased in HF-diet-fed rats showing a phenomenon of fatty liver, which could be significantly inhibited by treatment with RMD or orlistat.

Hepatic de novo lipogenesis is known as a procedure for the synthesis of fatty acid chains from acetyl-CoA subunits during glycolysis. The enzymes of ACC and FAS are involved in hepatic de novo lipogenesis, which contributes to the pathogenesis of NAFLD [26]. Moreover, NAFLD has also been found to be associated with the increased activity of HMGCR, a cholesterol synthesis rate limiting step enzyme [27]. In the present study, increased activities of ACC, FAS, and HMGCR in the livers of HF-diet-fed rats were found, which further enhanced the synthesis of TC and TG and lipid accumulation in the livers. Treatment with orlistat or RMD significantly reduced the HF diet feeding-induced dysregulation of these enzyme activities, TC and TG levels, and lipid accumulation in the livers. These results indicate that RMD may have the potential to prevent NAFLD.

The cumulative evidence reveals that the activation of AMPK induces a decrease in fatty acid synthesis, and reduces malonyl-CoA levels and enhances CPT-1 activity, resulting an increase in fatty acid oxidation in the liver [28]. Exercise has been found to induce a reduction in the level of malonyl-CoA through an AMPK-inactivated ACC signaling pathway [29]. Activation of AMPK could also inhibit PPAR-γ expression and reduce lipogenesis [30,31]. AMPK activation has been demonstrated to induce fatty acid oxidation in skeletal muscle through the activation of PPAR-α [32]. Moreover, a potent PPARα agonist has been found to stimulate hepatic mitochondrial CPT-1 expression [33]. Oral administration of orlistat to HF-diet-fed mice has been shown to reverse the HF diet downregulated AMPK phosphorylation in the liver [34]. In the present study, to understand the mechanism of RMD on hepatic lipid metabolism, we tested the signals of AMPK and its downstream molecules. We found that the protein expression of phosphorylated AMPK, PPAR-α, and CPT-1 was decreased, and the protein expression of PPAR-γ was increased in the liver of HF-diet-fed rats, which could be reversed by the treatment of RMD or orlistat. These findings indicate that RMD protects against HF diet feeding-induced fatty liver in response to AMPK activation.

## 5. Conclusions

Supplementation of RMD to HF-diet-fed rats can (1) inhibit liver lipid-synthesis-related enzymes ACC and FAS activity and cholesterol-synthesis-related enzyme HMGCR activity, which reduces the liver TC and TG levels; (2) activate AMPK phosphorylation and upregulate CPT1-α protein expression, which increases the fatty acid β-oxidation in the liver; (3) activate AMPK phosphorylation and downregulate PPAR-γ protein expression, which reduces liver lipid accumulation. Based on these findings, RMD has the beneficial effect of reducing hepatic lipid accumulation, and may have the potential to prevent progression of the disease from fatty liver.

## Figures and Tables

**Table 1:** Composition of experimental diets (%).

Ingredient (%)	NC	HF	HO	FS
Corn starch	64.8	49.1	49.1	49.1
Casein	20	20	20	20
Lard	3	18	18	18
Soybean oil	2	2	2	2
Vitamin ^1^	1	1	1	1
Mineral ^2^	4	4	4	4
Cholesterol	-	0.5	0.5	0.5
Cholic acid	-	0.2	0.2	0.2
Choline chloride	0.2	0.2	0.2	0.2
Cellulose	5	5	5	-
Fibersol-2	-	-	-	5
Orlistat	-	-	0.2	-
Total calories (kcal/100 g)	394.2	466.4	466.4	466.4
Carbohydrate (% kcal)	68.29	44.25	44.25	44.25
Protein (% kcal)	20.29	17.15	17.15	17.15
Fat (% kcal)	11.42	38.6	38.6	38.6

^1^ AIN-93 vitamin mixture; ^2^ AIN-93 mineral mixture; NC: normal control diet; HF: high-fat diet; HO: high-fat diet containing 0.2% orlistat; FS: high-fat diet containing 5% Fibersol-2.

**Table 2:** The changes of body weight and food and water intakes in high fat (HF)-diet-fed rats supplemented with or without orlistat and resistant maltodextrin (RMD) for 8 weeks.

Parameters	NC	HF	HO	FS
**Body weight (g)**	616.2 ± 22.5	638.0 ± 33.6 *	552.5 ± 35.6 **^,##^	616.5 ± 43.8
**Body weight gain (g)**	115.5 ± 14.7	107.2 ± 16.6	34.3 ± 15.2 **^,##^	106.4 ± 22.5
**Food intake (g/day)**	31.2 ± 5.2	21.5 ± 0.8 **	25.5 ± 2.0 *^,##^	20.9 ± 1.5 *
**Feed efficiency (%) ^1^**	3.9 ± 0.7	4.9 ± 0.7 *	1.5 ± 0.7 **^,##^	5.1 ± 0.7 *
**Water intake (mL/day)**	30.0 ± 6.5	28.0 ± 12.6	40.9 ± 6.9 **^,#^	28.3 ± 11.5

Results are expressed as mean ± standard deviation (S.D.) (*n* = 8 rats per group). * present *p* < 0.05, ** present *p* < 0.01 as compared with NC group; ^#^ present *p* < 0.05, ^##^ present *p* < 0.01 as compared with HF group. ^1^ Feed efficiency (%) = (body weight gain (g)/food intake (g/day)) × 100%. NC: normal control diet; HF: high-fat diet; HO: high-fat diet containing 0.2% orlistat; FS: high-fat diet containing 5% Fibersol-2.

**Table 3:** The changes of liver and adipose tissue weights in HF-diet-fed rats supplemented with or without orlistat and RMD for 8 weeks.

Parameters	NC	HF	HO	FS
**Liver weight (g)**	17.0 ± 1.4	36.2 ± 2.9 **	28.3 ± 4.5 **^,##^	33.5 ± 3.4 **
**Relative liver weight (g/100 g BW)**	2.7 ± 0.2	5.8 ± 0.5 **	5.0 ± 0.4 **^,##^	5.3 ± 0.4 **
**Adipose tissue weight (g)**	32.0 ± 4.7	28.5 ± 7.5	19.7 ± 7.4 **^#^	29.5 ± 6.2
**Relative adipose tissue weight (g/100 g BW)**	5.2 ± 0.7	4.5 ± 1.0	3.4 ± 1.0 *^#^	4.7 ± 0.8

Results are expressed as mean ± S.D. (*n* = 8 rats per group). * present *p* < 0.05, ** present *p* < 0.01 as compared with NC group; ^#^ present *p* < 0.05, ^##^ present *p* < 0.01 as compared with HF group. NC: normal control diet; HF: high-fat diet; HO: high-fat diet containing 0.2% orlistat; FS: high-fat diet containing 5% Fibersol-2; BW: body weight.

**Table 4:** The changes of plasma lipids and liver functional markers in HF-diet-fed rats supplemented with or without orlistat and RMD for 8 weeks.

Parameters	NC	HF	HO	FS
**Triglyceride (mg/dL)**	137.9 ± 44.6	23.1 ± 7.5 **	60.9 ± 21.8 **^,##^	27.9 ± 9.0 **
**Total cholesterol (mg/dL)**	90.1 ± 9.3	115.6 ± 16.2 *	117.3 ± 10.9 **	77.6 ± 14.7 ^#^
**HDL-C (mg/dL)**	54.2 ± 9.6	42.2 ± 10.0 *	28.8 ± 8.3 **^,#^	31.7 ± 5.4 **^,#^
**LDL-C (mg/dL)**	22.7 ± 6.6	55.0 ± 10.7 **	67.8 ± 13.0 **	32.1 ± 13.5 ^##^
**HDL-C/LDL-C ratio**	2.6 ± 0.9	0.8 ± 0.3 **	0.4 ± 0.2 **^,#^	1.2 ± 0.5 **
**AST (U/L)**	15.6 ± 2.0	82.2 ± 36.0 **	47.4 ± 21.2 **^,#^	50.8 ± 18.0 **^,#^
**ALT (U/L)**	8.6 ± 3.5	37.4 ± 5.9 *	29.3 ± 13.9 **^,#^	25.3 ± 8.9 **^,#^

Results are expressed as mean ± S.D. (*n* = 8 rats per group). * present *p* < 0.05, ** present *p* < 0.01 as compared with NC group; ^#^ present *p* < 0.05, ^##^ present *p* < 0.01 as compared with HF group. NC: normal control diet; HF: high-fat diet; HO: high-fat diet containing 0.2% orlistat; FS: high-fat diet containing 5% Fibersol-2; HDL-C: high-density lipoprotein cholesterol; LDL-C: low-density lipoprotein cholesterol; AST: aspartate transaminase; ALT: alanine aminotransferase.

**Table 5:** The changes of triglyceride and total cholesterol levels in the livers of HF-diet-fed rats supplemented with or without orlistat and RMD for 8 weeks.

Parameters	NC	HF	HO	FS
**Total cholesterol (mg/g liver)**	3.8 ± 2.3	138.4 ± 25.5 **	101.4 ± 16.5 **^,#^	119.7 ± 17.0 **^,#^
**Triglyceride (mg/g liver)**	14.0 ± 3.9	75.2 ± 10.2 **	41.6 ± 10.7 **^,##^	57.8 ± 15.5 **^,#^

Results are expressed as mean ± S.D. (*n* = 8 rats per group). * present *p* < 0.05, ** present *p* < 0.01 as compared with NC group; ^#^ present *p* < 0.05, ^##^ present *p* < 0.01 as compared with HF group. NC: normal control diet; HF: high-fat diet; HO: high-fat diet containing 0.2% orlistat; FS: high-fat diet containing 5% Fibersol-2.

**Table 6:** The changes of fecal weight, triglyceride, and total cholesterol levels of HF-diet-fed rats supplemented with or without orlistat and RMD for 8 weeks.

Parameters	NC	HF	HO	FS
**Fecal wet weight (g/day)**	2.0 ± 0.2	1.9 ± 0.2	5.7 ± 0.7 **^,##^	1.4 ± 0.2 **^,##^
**Fecal dry weight (g/day)**	1.9 ± 0.2	1.7 ± 0.1 *	4.0 ± 0.4 **^,##^	1.0 ± 0.1 **^,##^
**Triglyceride (mg/g feces)**	2.5 ± 1.4	3.1 ± 0.7 *	138.1 ± 46.8 **^,##^	4.7 ± 0.9 **^,##^
**Total cholesterol (mg/g feces)**	2.4 ± 0.6 ^a^	12.8 ± 3.1 **	13.9 ± 2.1 **	24.6 ± 4.1 **^,##^

Results are expressed as mean ± S.D. (*n* = 8 rats per group). * present *p* < 0.05, ** present *p* < 0.01 as compared with NC group; ^#^ present *p* < 0.05, ^##^ present *p* < 0.01 as compared with HF group. NC: normal control diet; HF: high-fat diet; HO: high-fat diet containing 0.2% orlistat; FS: high-fat diet containing 5% Fibersol-2.
